# “End-to-End Chromosome Fusion” as the Main Driver of Descending Dysploidy in *Vigna lasiocarpa* (Mart. ex Benth.) Verdc. (Leguminosae Juss.)

**DOI:** 10.3390/plants14121872

**Published:** 2025-06-18

**Authors:** Lazaro Serafim, Jarbson Henrique Silva, Sibelle Dias, Ana Rafaela da Silva Oliveira, Maria Clara Nunes, Antônio Félix da Costa, Ana Maria Benko-Iseppon, Jiming Jiang, Lívia do Vale Martins, Ana Christina Brasileiro-Vidal

**Affiliations:** 1 Departamento de Genética, Centro de Biociências, Universidade Federal de Pernambuco, Recife 50670-901, Pernambuco, Brazil; lazaro.serafim@ufpe.br (L.S.); jarbson.silva@ufpe.br (J.H.S.); sibelle.dias@ufpe.br (S.D.); oliveiraars.bio@gmail.com (A.R.d.S.O.); mclaravn16@gmail.com (M.C.N.); ana.iseppon@gmail.com (A.M.B.-I.); 2 Instituto Agronômico de Pernambuco, Recife 50761-000, Pernambuco, Brazil; felix.antonio@ipa.br; 3 Department of Plant Biology, Michigan State University, East Lansing, MI 48824, USA; jiangjm@msu.edu; 4 Universidade Federal do Piauí, Floriano 64800-000, Piauí, Brazil; liviavalem@ufpi.edu.br

**Keywords:** oligo-FISH, *Lasiospron*, dysploid species, chromosome fusion

## Abstract

The genus *Vigna* Savi (Leguminosae Juss.) comprises approximately 150 species, classified into five subgenera, most of which exhibit a diploid chromosome number of 2*n* = 22. However, the wild species *Vigna lasiocarpa* (Benth) Verdc. (*V*. subg. *Lasiospron*) is notable for its dysploid chromosome number of 2*n* = 20. This study aimed to elucidate the chromosomal events involved in the karyotype evolution of *V. lasiocarpa* (*Vla*). We used oligopainting probes from chromosomes 1, 2, 3, and 5 of *Phaseolus vulgaris* L. and two barcode probes from the genome of *V. unguiculata* (L.) Walp. Additionally, bacterial artificial chromosomes (BACs) from *V. unguiculata* and *P. vulgaris*, along with a telomeric probe from *Arabidopsis thaliana* (L.) Heynh., were hybridized to *V. lasiocarpa* metaphase chromosomes to characterize *Vla*3, *Vla*7/5, and *Vla*9. Our findings revealed conserved oligo-FISH patterns on chromosomes 2, 6, 8, 10, and 11 between *V. unguiculata* and *V. lasiocarpa.* Paracentric and pericentric inversions were identified for *Vla*3 and *Vla*9, respectively. Our integrative approach revealed that the dysploid chromosome originated from an “end-to-end fusion” of homoeologous chromosomes 5 and 7. This is the first report on the chromosomal mechanisms underlying descending dysploidy in *Vigna*, providing new insights into the evolutionary dynamics of the genus.

## 1. Introduction

Changes in chromosome number and structure are major drivers of species evolution and diversification, leading to disruptions in collinearity and/or synteny among related species [1]. The two primary biological mechanisms responsible for alterations in chromosome number within a karyotype are aneuploidy and dysploidy. Aneuploidy refers to the gain or loss of whole chromosome(s), whereas dysploidy involves changes in chromosome number, either an increase (ascending dysploidy) or decrease (descending dysploidy), resulting from chromosomal rearrangements, such as fissions and fusions, without a net gain or loss of the genetic material [2]. In plants, dysploidy is a common evolutionary event that has shaped the genomes of various species, as evidenced in several angiosperm families, such as Brassicaceae, Asteraceae, and Solanaceae [3].

The paraphyletic *Vigna* Savi genus belongs to the Leguminosae Juss. family and the Papilionoideae subfamily. It comprises approximately 150 accepted species with a worldwide distribution (https://www.worldfloraonline.org/). Closely related to *Phaseolus* L., *Vigna* diverged approximately 10.4 million years ago [4]. This genus includes several socioeconomically and nutritionally important grain legumes, such as cowpea [*V. unguiculata* (L.) Walp.], mung bean (*V. radiata* L.), and adzuki bean [*V. angularis* (Willd.) Ohwi and Ohashi] [5,6,7].

*Vigna* is divided into five subgenera, each with distinct centers of origin: *Lasiospron* (Benth.) Maréchal, Mascherpa & Stainier (American); *Ceratotropis* (Piper) Verdc. (Asian); and *Vigna*, (R.Wilczek) Verdc., *Plectrotropis* (Schumach.); and *Haydonia* (R.Wilczek) Verdc. (African) [8,9,10]. The monophyletic *Vigna* subg. *Lasiospron* consists of six wild species native to the wet tropical forests of the Americas, characterized by distinct morphological features compared to other *Vigna* species [9]. This early-diverging subclade of *Vigna* subg. *Lasiospron* has been identified as a sister group to the remaining Old Word *Vigna* clade (*Vigna* sensu stricto), with an estimated divergence time of approximately 4–5 million years (My) [8,9]. Most *Vigna* species have a stable diploid karyotype of 2*n* = 22 [11,12,13]. However, a reduction in chromosome number has been documented in 6 of the 150 *Vigna* species (https://taux.evolseq.net/CCDB_web; accessed on 10 February 2025). Among them, *Vigna lasiocarpa* (Benth.) Verdc. (syn. *Phaseolus pilosus* Kunth.) stands out as the only dysploid species reported within the small *Vigna* subg. *Lasiospron*, possessing a karyotype of 2*n* = 2x = 20 [9,14].

Since its development, oligonucleotide fluorescent in situ hybridization (oligo-FISH) technique has emerged as a powerful and versatile tool in plant cytogenetics [15]. It has been widely applied in various studies, including comparative karyotype analysis [16], chromosome behavior during meiosis [17], identification of chromosomal territories in nuclei [18], macrosynteny analysis [19], identification of inter- and intraspecific hybrid chromosome [20,21], genome assembly validation [22], and investigation of chromosome number reduction in dysploid species [23]. Our extensive research on *Vigna* species has revealed that chromosomal inversions and translocations are the primary forces driving evolutionary divergence among *Vigna* subgenera. We have used various probe sets, including bacterial artificial chromosomes (BACs), 5S and 35S ribosomal DNA (rDNA), and oligo probes [24,25,26,27]. However, dysploid *Vigna* species have received little attention, making them an excellent target for investigating the evolutionary pathway associated with chromosome reduction.

We performed a cytomolecular mapping in the dysploid *species V. lasiocarpa* using a well-established methodology based on chromosome painting and barcode oligo-FISH, along with BAC probes from *P. vulgaris* and *V. unguiculata*. Our results reveal that “end-to-end chromosome fusion” (EEF) is the primary mechanism driving chromosome reduction in *V. lasiocarpa* species. Our findings provide valuable insights into the key chromosomal rearrangements underpinning speciation in *V. lasiocarpa*, enhancing our understanding of the diversity and evolution of this important group of legume crops.

## 2. Results

### 2.1. Chromosome Mapping and rDNA Sites in V. lasiocarpa

**Figure 1:**
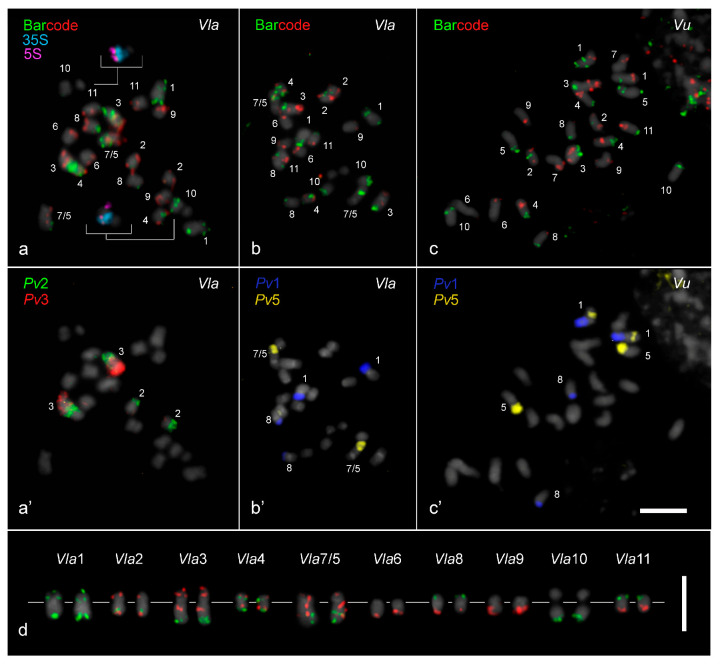
Oligo-FISH and chromosome painting based karyotyping of *Vigna lasiocarpa* (2*n* = 20) and *V. unguiculata* (2*n* = 22). (**a**) Barcode probes hybridized to *V. lasiocarpa* chromosomes, (**a’**) followed by a re-hybridization with *P. vulgaris* painting probes for chromosomes 2 (*Pv*2, green) and 3 (*Pv*3, red), enabling the identification of their putative homoeologs in *V. lasiocarpa*. (**a**) Both 5S (pseudo-colored in magenta) and 35S (pseudocolored in light blue) rDNA sites were mapped to a single chromosome *Vla*10, highlighted in the inserts. (**b**) The barcode probes hybridized to *V. lasiocarpa* chromosomes, (**b’**) followed by a re-hybridization with *Pv*1 (dark blue) and *Pv*5 (yellow) probes, allowing the identification of homoeologous chromosomes *Vla*1, *Vla*8, and the dysploid *Vla*7/5 chromosome. (**c**) Barcode probes hybridized to *V. unguiculata* chromosomes, (**c’**) followed by a re-hybridization using *Pv*1 (dark blue) and *Pv*5 (yellow) probes. All chromosomes were counter-stained with DAPI (pseudocolored in gray). (**d**) Karyogram of the barcode pattern in *V. lasiocarpa* (*Vla*), with the centromeres aligned along a gray line. Bars in (**c’**,**d**) = 5 µm.

The two barcode pools developed for *V. unguiculata* and the painting probes of *P. vulgaris* chromosomes 1, 2, 3, and 5 were hybridized to the 10 chromosome pairs of *V. lasiocarpa* (Figure 1a,a’,b,b’). Additionally, the barcode probes and the *Pv*1 and *Pv*5 painting probes were used to hybridize the *V. unguiculata* karyotype (Figure 1c,c’), showing a translocation event involving chromosomes 1 and 5 that was not observed in *V. lasiocarpa*. The painting probes exhibited strong signals on chromosomes from both *Vigna* species, except in the pericentromeric and centromeric regions, probably due to sequences divergence in these areas (Figure 1a’–c’).

Both 5S and 35S rDNA in *V. lasiocarpa* were each mapped to a single site on chromosome *Vla*10 (inserts in Figure 1a). The 5S rDNA site was localized at the subterminal region on the long arm, while the 35S rDNA site was localized in a proximal region at the same arm. The chromosome numbering of *V. lasiocarpa* (*Vla*) was consistent with the homologous chromosomes of *V. unguiculata* (*Vu*) (Figure 1d).

A similar FISH signal pattern for both barcode and painting probes was observed on chromosomes 2, 6, 8, 10, and 11 from *V. unguiculata* and *V. lasiocarpa* (Figure 1). For instance, painting probes derived from *P. vulgaris* chromosomes 2 (*Pv*2, green) and 3 (*Pv*3, red), when hybridized to the *V. lasiocarpa* karyotype, produced the expected labeling pattern for chromosome 2, as previously observed in seven other *Vigna* species/subspecies [27]. Chromosome *Vla*2 exhibited signals at the distal region of the short arm labeled with *Pv*3 (red) and the distal region of the long arm labeled with *Pv*2 (green), with its pericentromeric region lacking the painting signals (Figure 1a’,c’). It is important to note that the reciprocal translocation *Pv*2/*Pv*3 occurred in *P. vulgaris* and was not shared with *Vigna* species (Figure 1a’).

**Figure 2:**
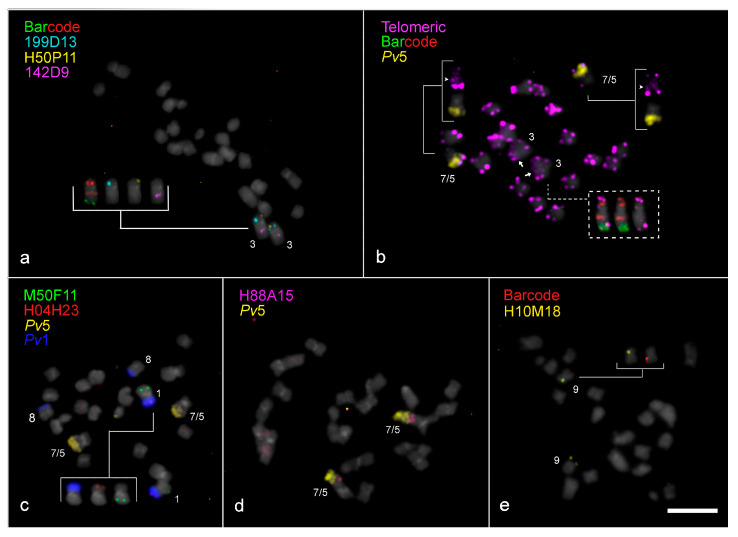
Chromosomal rearrangements in *Vigna lasiocarpa* (*Vla*, 2*n* = 20) revealed by FISH. (**a**) A paracentric inversion involving chromosome *Vla*3, identified by re-hybridization of BACs 199D13 (light blue, from *Pv*3L), 142D9 (magenta, from *Pv*3S), and H50P11 (yellow, from *Vu*3L). From left to right, the insert shows the homoeologous *Vla*3 hybridized with the barcode probes, followed by the above-mentioned BACs. (**b**) A metaphase cell of *V. lasiocarpa* hybridized with a telomeric DNA probe (magenta) and re-hybridized with *Pv*5 (yellow) and the barcode probes, highlighting the “end-to-end chromosome fusion” in the dysploid *Vla*7/5. The arrowheads point to the interstitial telomeric site on *Vla*7/5 (upper inserts), while the arrows point to the interstitial telomeric site in *Vla*3, indicating a putative inversion. The lower dotted insert shows chromosomes from a different metaphase cell hybridized with telomeric and barcode probes. (**c**) Reciprocal translocation between chromosomes *Pv*1 (dark blue) and *Pv*5 (yellow), confirmed by BAC M050F11 (green, *Vu*5L) and BAC H04H23 (red, *Vu*1L), hybridized to *Vla*1 and *Vla*8. From left to right, the insert shows homoeologous *Vla*1 hybridized with the *Pv*1 oligopainting probe followed by the above-mentioned BACs. (**d**) Confirmation of the *Vla*7/5 chromosomal fusion using FISH with *Pv*5 (yellow) and BAC H088A15 (magenta, *Vu*7S) probes. (**e**) A pericentric inversion in chromosome *Vla*9, identified by FISH with barcode and BAC H010M18 (yellow, *Vu*9L) probes, showing terminal localization of the BAC signal. All chromosomes were counterstained in DAPI and pseudocolored in gray. Bar in (**e**) = 5 µm.

**Figure 3:**
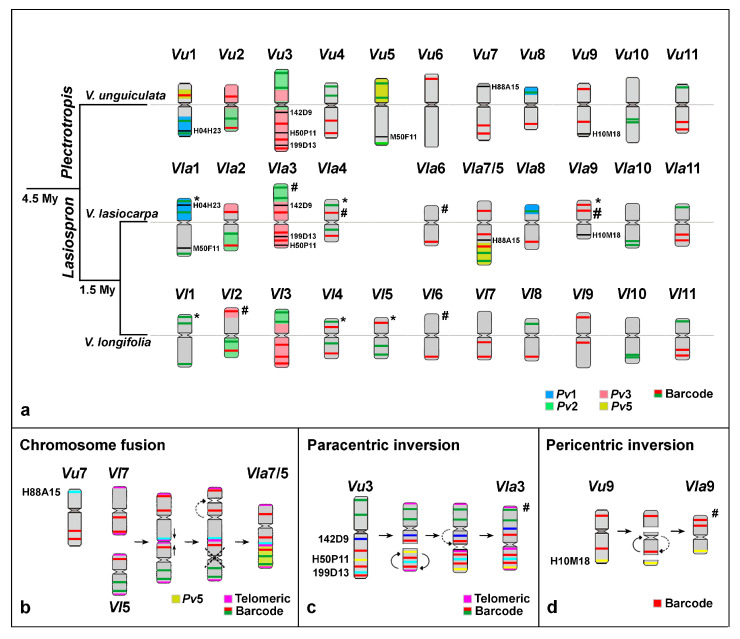
Phylogenetic relationships among *Vigna unguiculata* (*Vu*), *V. lasiocarpa* (*Vla*), and *V. longifolia* (*Vl*), highlighting major chromosomal rearrangements associated with the evolution of *V. lasiocarpa*. (**a**) Idiograms of *V. unguiculata* (subgenus *Plectotropis*), *V. lasiocarpa*, and *V. longifolia* (both from subgenus Lasiospron) are plotted along a phylogenetic tree, based on Delgado-Salinas et al. [9] and Horton et al. [10]. The divergence times between the two subgenera and between *V. lasiocarpa* and *V. longifolia* are indicated along the branches. The barcode signal patterns and the *Pv*2 and *Pv*3 painting in *V. unguiculata* followed de Oliveira Bustamante et al. [26] and do Vale Martins et al. [25], respectively, while data for *V. longifolia* followed Dias et al. [27]. The chromosomes are labeled using the species abbreviation and their respective chromosome numbering. Barcode (red and green bars), BACs H50F11 and H04H23 (black bars), and painting probes *Pv*1, *Pv*2, *Pv*3, and *Pv*5 (light blue, green, red, and yellow, respectively) are mapped onto the idiograms of each *Vigna* species. Centromeres are aligned by a gray line. Asterisks (*) on *Vla*1, *Vla*4, *Vla*9, *Vl*1, *Vl*4, and *Vl*5 indicate changes in barcode patterns compared to *V. unguiculata*. Hashtags (#) on *Vl*2, *Vla*3, *Vla*4, *Vla*6, *Vl*6, and *Vla*9 indicate changes in chromosome arm orientation compared to *V. unguiculata*. (**b**) Schematic representation of the “end-to-end chromosome fusion” event involving *Vla*5 and *Vla*7, evidenced by barcode (red and green), telomeric (magenta), BAC H088A15 (light blue), and the *Pv*5 (yellow) probes. A black dotted X marks the position of the centromere, which was not visible after the chromosome fusion. The dottle arrow indicates a possible centromere shift following the “chromosome fusion”. (**c**) Paracentric inversion on *Vla*3, inferred from barcode and telomeric probe signals (magenta), along with BAC 142D9 (blue), H50P11 (yellow), and 199D13 (light blue), indicating the inversion of an interstitial region on *Vla*3L. The dottle arrow indicates a possible centromere shift after the rearrangement. The centromere, previously flanked by one green barcode signal and the BAC 142D9 (blue), is now located between one red barcode signal and the proximal telomeric probe. (**d**) Pericentric inversion on *Vla*9, with the barcode probe and the BAC H010M18 (yellow) showing both red barcode signals located adjacently on *Vla*9L as a result of the inversion.

In contrast, distinct FISH signal patterns were observed on the homoeologous *V. unguiculata* chromosomes 1, 3, 4, and 9, and particularly for chromosomes 5 and 7 (Figure 2 and Figure 3b–d). For example, the barcode FISH signal pattern on *Vla*4 was similar to *Vl*4, with one red and one green signal on each chromosome arm, but different from *Vu*4, which exhibited two green signals on the short arm and two red signals on the long arm (Figure 1a–c and Figure 3a). This pericentric inversion appears to be exclusive to *V. unguiculata* so far. The rearrangements involving the remaining chromosomes, which likely contributed to the genomic diversification and speciation of *V. lasiocarpa*, are illustrated schematically in Figure 3 and are further detailed in the following sections. The chromosomal positions of the probes in the three species are presented in Supplementary Table S1.

### 2.2. Translocation Among Vigna Chromosomes 1, 5, and 8

Painting probes from *P. vulgaris* chromosomes 1 and 5 hybridized to three chromosome pairs (1, 5, and 8) in both *V. lasiocarpa* and *V. unguiculata* (Figure 1b’,c’). In *V. unguiculata*, the *Pv*1 probe (blue) distally labeled more than half of *Vu*1L and almost half of *Vu*8S, while the *Pv*5 probe (yellow) labeled the entire short arm of *Vu*5 and an interstitial portion of *Vu*1S. These findings confirm the presence of a translocation involving chromosomes 1 and 5 in *V. unguiculata*, which is absent in other *Vigna* species. We point out that the translocation *Pv*1/*Pv*8 has occurred in *Phaseolus* but not in *Vigna* species (Figure 1a’ and Figure 3a).

In *V. lasiocarpa*, the *Pv*1 probe (blue) hybridized to two chromosome pairs: the entire *Vla*1S and the distal half of *Vla*8S. Conversely, the *Pv*5 probe (yellow) distally labeled almost half of the long arm of the larger chromosome pair (*Vla*7/5) (Figure 1b’ and Figure 3a). The *Pv*1/8 translocation was confirmed by the *V. lasiocarpa* pattern. Additionally, the *Vu*1/5 reciprocal translocation was absent in *V. lasiocarpa*. This finding was confirmed by using the BAC H050F11 (*Vu*5L), which was located on *Vla*1L, and the BAC H004H23 (*Vu*1L), located on *Vla*1S (Figure 2c and Figure 3a). Based on *Pv*1 and *Pv*5 chromosome painting and barcode patterns, we revised the previously proposed homoeology of *V. longifolia* chromosomes 1 and 5, as reported by [27]. The assignments have been corrected, with the former chromosome 1 now designated as *Vl*5 and the former chromosome 5 as *Vl*1.

### 2.3. Chromosomal Rearrangements Associated with the Evolution of V. lasiocarpa

#### 2.3.1. Paracentric Inversion on Vla3 Confirmed by the Presence of an Additional Interstitial Telomeric Signal

A paracentric inversion was observed on *Vla*3 when compared to *Vl*3 in addition to a distinct *Vla*3 orientation. This chromosome exhibited the same barcode pattern as *Vu*3 and *Vl*3, with two green and three red signals across *Vla*3, but with centromere positioned between two red barcode marks, instead of one green and one red mark, as observed in the other *Vigna* species (Figure 1a,c and Figure 3a). Additionally, the short arm and the proximal region of the long arm of *Vla*3 were labeled with *Pv*3 (red), while the distal portion of the long arm was labeled with *Pv*2 (green), with the centromere embedded in *Pv*3 sequences. On the other hand, *V. longifolia* exhibited the same painting pattern, but mapped to opposite chromosome arms, supporting the inversion event inferred in *V. lasiocarpa* (Figure 3a). Moreover, the oligo-FISH pattern of *V. lasiocarpa* reflects a morphological change in *Vla*3, as indicated by the hashtag in Figure 3a. This finding was unexpected, as paracentric inversions typically do not affect chromosome morphology.

To further investigate the structural organization of *Vla*3, three specific BAC probes were used: 199D13 (from *Pv*3L), 142D9 (from *Pv*3S), both syntenic with *Vu*3, and H50P11 (from *Vu*3L). BAC 199D13 hybridized to the short arm of *Vla*3 between two red barcode signals, while BAC 142D9 hybridized to the interstitial region of the long arm between a red and a green barcode signal, consistent with the expected pattern for *Vu*3 (Figure 3c). On the other hand, BAC H50P11 hybridized to the subterminal region of *Vla*3S, differing from its position in *Vu*3L, which was mapped to an interstitial position (Figure 2a and Figure 3c). These results support the occurrence of a paracentric inversion in *Vla*3, which reorganized the relative positions of these markers along the chromosome.

FISH using a telomeric DNA probe was performed to further understand this structural rearrangement in *Vla*3. In addition to the expected terminal signals on all *V. lasiocarpa* chromosome arms, an extra telomeric signal was detected in a proximal region of *Vla*3S, between the two red signals (Figure 2b and Figure 3c). This interstitial telomeric signal, along with the repositioning of BACs H50P11 and 199D13, provides strong evidence for the occurrence of a paracentric inversion on *Vla*3. However, we also observed a centromere shift in *Vla*3 compared to *Vu*3 and *Vl*3, with the *Vla*3 centromere positioned between two red barcode marks, now located near the terminal *Vu*3L BAC 199D13 instead of the proximal *Vu*3L BAC 142D9 (Figure 3c and Supplementary Figure S1). This shift altered the chromosome morphology and suggests a centromere repositioning besides the paracentric inversion. The dashed arrow in Figure 3c schematically illustrates this repositioning.

#### 2.3.2. “End-to-End Vla7/5 Chromosome Fusion” as the Major Driver for Descending Dysploidy in *V. lasiocarpa*

We used a combination of BAC, telomeric DNA, and oligo-FISH probes to further investigate the structure of the distinctively large dysploid chromosome in *V. lasiocarpa*. In the oligo-FISH barcode analysis, this chromosome showed a unique signal pattern: one red signal on the short arm and two red and two green signals on the long arm. In addition to the expected terminal sites, an interstitial telomeric signal was detected, suggesting a break at the ends of both chromosomes, followed by a reciprocal translocation. This rearrangement may have generated two “fused chromosomes” with the loss of the small chromosome, which was not observed in *V. lasiocarpa* cells. These alterations could explain the origin of the descending dysploidy. To facilitate our understanding of this chromosomal rearrangement, this reciprocal translocation will hereafter be referred to as “end-to-end chromosome fusion” (EEF). To confirm this rearrangement, we performed additional FISH experiments using a combination of painting and BAC probes (Figure 2d and Figure 3a,b). The distal half of the long arm was entirely labeled with the *Pv*5 probe (yellow), and BAC H88A15 (*Vu*7S) hybridized to an interstitial position on the same arm. These findings provide strong evidence that the descending dysploidy in *V. lasiocarpa* resulted from an EEF involving the homoeologous chromosomes 5 and 7 (Figure 3b).

The centromere in the dysploid chromosome was located between the two red barcode signals of the *Vla*7, rather than adjacent to both signals as observed in the ortholog chromosomes of *Vigna* species (Figure 3a,b). Additionally, the centromeric region expected for *Vla*5 was not detected in the fused chromosome (Figure 3b).

#### 2.3.3. Exclusive Pericentric Inversion on Vla9

In *Vla*9, a distinct barcode FISH pattern was observed when compared to *Vu*9, characterized by two red signals located on the same arm, whereas in *Vu*9, these signals are positioned on opposite arms (Figure 1a–c, Figure 2e and Figure 3d). This pattern is indicative of a pericentric inversion involving one of the red barcode signals in *Vla*9. To further investigate this rearrangement, we performed FISH using the BAC H10M18 (from *Vu*9L) in combination with the red barcode probe. While H10M18 hybridized to the *Vla*9S, both red barcode signals were detected in adjacent positions on *Vla*9L, supporting the inversion hypothesis. This represents the first report of a pericentric inversion on chromosome 9 in a *Vigna* species, indicating that it may be a lineage-specific chromosomal rearrangement unique to *V. lasiocarpa.*

## 3. Discussion

### 3.1. Chromosomal Rearrangements Associated with Descending Dysploidy and Evolution of V. lasiocarpa

Our research provides novel insights into the evolutionary mechanisms underlying descending dysploidy in *V. lasiocarpa*, a species belonging to *Vigna* subg. *Lasiospron*, one of the most basal subgenera within the *Vigna* lineage [10]. We identified three major chromosomal rearrangements associated with the diversification and speciation of this dysploid species: (1) “an end-to-end fusion of chromosomes” 5 and 7; (2) a paracentric inversion on chromosome 3; and (3) a pericentric inversion on chromosome 9. These structural changes appear to be exclusive to *V. lasiocarpa* and may represent lineage-specific events that occurred after the divergence of *Vigna* subg. *Lasiospron* and *Vigna* subg. *Plectrotropis*, which is estimated to have occurred approximately 4.5 Mya [9].

Inversions are among the most common chromosomal rearrangements in plants and have been associated with changes in gene expression, suppression of recombination, and genomic diversification [28]. Although their precise biological roles remain unclear, inversions are widely recognized as important drivers of genome evolution and speciation. For instance, several inversions, typically ranging from 100 to 200 kb, along with translocations, have contributed to the divergence between wild and cultivated soybean varieties [29]. Our recent findings reinforce the role of inversions and translocations in shaping the genome architecture and evolutionary diversification of *Vigna* species [25,27]. In the present work, a paracentric inversion on *Vla*3 with one interstitial and one terminal breakpoint was inferred based on the presence of additional Interstitial Telomeric Repeats (ITRs) and was confirmed through FISH using BAC probes from *P. vulgaris* and *V. unguiculata*. Previous studies have also reported rearrangements on chromosome 3 among *Vigna* and *Phaseolus* species [25,26,27,30], suggesting that this chromosome may represent a hotspot for rearrangements. Notably, in *Phaseolus*, ITRs associated with inversion events have already been described. In *P. microcarpus*, for example, an additional ITR signal was identified on chromosome 3, derived from a pericentric inversion involving the entire short arm and part of the long arm [31].

We also identified a telomeric probe signal at an interstitial position on the dysploid chromosome *Vla*7/5. The presence of ITRs, particularly in centromeric or pericentromeric regions, is a reliable marker of end-to-end fusions between non-homologous chromosomes [32,33,34,35]. In *V. lasiocarpa*, the fusion between chromosomes 5 and 7 was confirmed through FISH using a combination of a BAC probe from *Vu*7 and an oligopainting probe corresponding to *Vu*5, supporting the conclusion that this descending dysploidy arose via non-homologous chromosome fusion.

Dysploidy has played a key role in shaping chromosome number evolution across various plant species, as observed in the case of *V. lasiocarpa* in the present study. Mandáková et al. [36] demonstrated that end-to-end chromosome fusions are the primary mechanism driving independent descending dysploidy in the Microlepidieae (MICR) lineage. A comparable pattern is observed in the closely related *Phaseolus* genus, in which three dysploid species (*P. macvaughii*, *P. leptostachyus*, and *P. micranthus*) with 2*n* = 20 chromosomes have been identified within the Leptostachyus group [37,38]. Recent studies have revealed a high rate of chromosomal rearrangements in *P. leptostachyus* and *P. macvaughii*, including 16 rearrangements in *P. leptostachyus*, one of which involved a nested chromosome fusion [23]. The high frequency of chromosomal changes in the Leptostachyus group may be related to the abundance of repetitive elements, which are thought to have played a major role in the genomic restructuring and diversification of *Phaseolus* and related genera [39,40]. These observations highlight the importance of further investigation into the distribution and composition of repetitive DNA across *Vigna* genomes, particularly in dysploid species, to better understand how these elements influence genome architecture and evolution.

### 3.2. Positional Centromere Changes on V. lasiocarpa Chromosomes

The centromere plays a fundamental role in chromosome segregation and transmission by serving as the assembly site for the kinetochore, which mediates attachment to the mitotic spindle fibers, thereby ensuring accurate chromosome movement [41]. Despite this essential function, centromeres in eukaryotes display remarkable variation in size, structure, and sequence composition, a phenomenon known as centromere paradox. Their formation and identity are largely governed by epigenetic mechanisms [42,43]. Moreover, changes in centromere position without disrupting gene collinearity are frequently observed in plant genomes and can arise through different mechanisms, including (1) a pericentric inversion with breaks in the opposite arms—one near the centromere and the other distantly located—followed by a paracentric inversion involving the distal breakpoint and a new break adjacent to centromere; (2) a three-break-event, in which the centromeric fragment is inserted at a third breakpoint, distinct from the original centromere position; and (3) de novo centromere formation at a novel chromosomal location, generally accompanied by inactivation of the original centromere [44]. These mechanisms are referred to as centromere repositioning [44,45]. In the present study, we identified positional changes in the centromere of chromosomes *Vla*3, *Vla*9, and *Vla*7/5.

In cases of descending dysploidy, an end-to-end fusion (EEF) typically occurs between two non-homologous chromosomes. This fusion may be mediated by two DSBs at the terminal regions of both chromosomes, followed by a reciprocal ligation that results in the formation of a dicentric chromosome. To ensure proper chromosome segregation and maintain genome stability, one of the two centromeres must be subsequently silenced or eliminated [3,46]. We propose that such a mechanism underlies the formation of the dysploid chromosome 7/5 in *V. lasiocarpa*, in which the centromere of the ancestral chromosome 5 appears to have been inactivated, followed by a potential repositioning of the remaining functional centromere, stabilizing the fused chromosome.

On the other hand, the centromere shift on chromosome *Vla*9 is consistent with an unequal pericentric inversion. In turn, the centromere relocation on *Vla*3 was not a result of the paracentric inversion but rather appears to have resulted from a de novo centromere formation accompanied by the silencing of the ancestral centromere. Previous studies have reported centromere repositioning through de novo centromere formation in the *Vigna* genus [25,27]. This phenomenon appears to have occurred across multiple chromosomes in *Vigna* and *Phaseolus* species, based on analyses of genomic blocks (GBs) from *V. unguiculata* subsp. *unguiculata*, *V. unguiculata* subsp. *sesquipedalis*, and *P. vulgaris*, compared to the Ancestral Phaseoleae Karyotype (APK) [27,47]. Centromere repositioning has also been documented in other plant groups, such as the Brassicaceae family, particularly within the Arabideae tribe, where a high frequency of repositioning events has been observed. They are referred to as evolutionary new centromeres (ENCs) and can influence the epigenetic regulation of nearby genes [45].

Epigenetic mechanisms, such as CENH3/CENPA deposition, are believed to facilitate centromeric flexibility, enabling repositioning events in response to evolutionary and genomic pressures [48]. These processes not only contribute to chromosomal stability but may also play a significant role in plant speciation. To advance our understanding of centromere dynamics in *Vigna*, further functional and comparative studies, such as chromatin immunoprecipitation sequencing (ChIP-seq) or fine-scale centromere mapping, are essential to unravel the mechanisms and evolutionary implications of centromere repositioning in this genus.

### 3.3. Comparative Analysis Between Two Species of the American subg. Lasiospron: V. longifolia and V. lasiocarpa

Two of the six species from the American *Lasiospron* subgenus analyzed in this study, *V. longifolia* and *V. lasiocarpa*, are native to the Amazon basin, Restinga, and the Paraguay-Paraná river system [9]. These species are phylogenetically and morphologically closely related, both characterized by nearly cylindrical pods with thickened walls and subglobose seeds. This contrasts with other American *Vigna* species, which typically bear laterally flattened pods and seeds [9,10]. Despite their similarities, slight morphological differences distinguish *V. lasiocarpa* from *V. longifolia*. For instance, *V. lasiocarpa* generally presents five to seven floral nodes per inflorescence and a short-hooked style, whereas *V. longifolia* typically has two to three floral nodes and a poorly developed style [9].

Cytogenetically, chromosomal changes have occurred between *V. lasiocarpa* and *V. longifolia*, as evidenced by breaks in collinearity and macrosynteny involving five chromosomes. Additionally, *V. lasiocarpa* has undergone evolutionary processes leading to descending dysploidy (2*n* = 20), in contrast to *V. longifolia* and most *Vigna* species, which retain the ancestral chromosome number of 2*n* = 22 [49,50,51,52]. Our previous cytogenetic analyses revealed that *V. longifolia* possesses a conserved karyotype compared to other *Vigna* species, with only minor differences in the painting patterns of chromosomes 2 and 3 [27]. In contrast, the major chromosomal rearrangements observed in *V. lasiocarpa*, including a paracentric inversion on chromosome 3, a pericentric inversion on chromosome 9, and an “end-to-end fusion” between chromosomes 5 and 7, are likely species-specific events that occurred after the divergence between *V. lasiocarpa* and *V. longifolia*, estimated at approximately 1.5 Mya [9].

Taken together with our previous studies involving species from different origins and subgenera, our findings provide additional evidence that deepens our understanding of the mechanisms shaping *Vigna* karyotypes and their implications for biodiversity and speciation. Therefore, further research on other understudied species is needed to develop a broader overview of the karyotype evolution and genome organization in these important *Vigna* legumes.

## 4. Materials and Methods

### 4.1. Plant Material and Chromosome Preparation

Seeds of *V. unguiculata* cv. BR-14 Mulato and *V. lasiocarpa* (PI 306376) were obtained from Embrapa Meio-Norte (Teresina, Piauí, Brazil) and the U.S. National Plant Germplasm System (NGPS, Odessa, TX, USA), respectively, and were multiplied at the Instituto Agronômico de Pernambuco (IPA, Recife, Brazil).

Root tips from germinated seeds were pretreated with 8-hydroxyquinoline solution (2 mM) for 5 h at 18 °C, then fixed in a solution of 3:1 (*v*/*v*) methanol: 1 glacial acetic acid for 2–22 h at room temperature (RT) and stored at −20 °C until use. For chromosome preparation, the root tips were washed twice in distilled water and digested in an enzymatic solution consisting of 2% pectolyase (*w*/*v*), 4% cellulase (*w*/*v*), and 20% pectinase (*v*/*v*) for 2 h at 37 °C in a moist chamber. After the digestion, the slides were prepared using the air-drying protocol [53] with minor modifications: after air-drying, the slides were incubated in 45% acetic acid for 2 min (RT) and dried for 5 min at 37 °C.

### 4.2. FISH Probe Sets

To identify the individual chromosomes and the chromosomal rearrangements between *V. lasiocarpa* and *V. unguiculata*, we performed the FISH technique using various oligo probes from *V. unguiculata* and *P. vulgaris* (oligobarcode and chromosome painting, respectively), as well as BAC probe sets from both species, rDNA, and telomeric probes.

For chromosome painting, the probes were designed from *P. vulgaris* genome (Pvulgaris_442_v2.0; available at https://phytozome.jgi.doe.gov/), for chromosomes 2 and 3 (*Pv*2 and *Pv*3), as described by do Vale Martins et al. [25], and for chromosomes 1 and 5 (*Pv*1 and *Pv*5), as described by Montenegro et al. [54]. For the barcode probes, we used two libraries designed from the *V. unguiculata* reference genome (‘IT97K-499-35’, [55]), as described by de Oliveira Bustamante et al. [26]. Barcode libraries 1 (red) and 2 (green) correspond to 16 and 14 FISH signals, respectively, covering the 11 chromosome pairs of different *Vigna* species [27]. The oligo probes were synthesized by Arbor Biosciences (Ann Arbor, MI, USA), amplified, and indirectly labeled with biotin or dual biotin (www.idtdna.com, *Pv*1, *Pv*2, and library 2) and digoxigenin (www.idtdna.com, *Pv*3, *Pv*5, and library 1) according to published protocols [18,56].

Five BAC clones of *V. unguiculata* chromosomes (*Vu*) were selected: *Vu*1L (H004H23, long arm of *Vu*1), *Vu*3L (H050P11), *Vu*5L (M050F11), *Vu*7S (H088A15; short arm of *Vu*7), and *Vu*9L (H010M18). These BACs were previously mapped in *V. unguiculata* mitotic chromosomes by do Vale Martins et al. [25]. Additionally, we selected BACs from *Pv*3S and *Pv*3L of *P. vulgaris* (142D9 and 199D13, respectively), which were previously mapped in *P. vulgaris* by Pedrosa-Harand et al. [57]. The BAC synteny analysis between *V. unguiculata* and *P. vulgaris* was deeply described by de Oliveira Bustamante et al. [26]. All BACs were tested and used to confirm the chromosomal rearrangements between *V. unguiculata* and *V. lasiocarpa*. Moreover, we used the D2 sequence, a 400 bp fragment of two 5S rDNA repeat units from Lotus japonicus (Regel) K. Larsen [58], and the R2 sequence, a 6.5 kb fragment of 18S-5.8S-25S rDNA repeat unit from *Arabidopsis thaliana* [59]. The *Arabidopsis thaliana* plasmid clone, pAtT4 [60], served as a telomeric (5′-CCCTAAA-3′) DNA probe. BAC, 5S, 35S rDNA, and pAtT4 clones were isolated using the Plasmid Mini Kit (Qiagen). The BAC clones were directly labeled with Cy3-dUTP (GE Healthcare). The telomeric and 35S rDNA probes were indirectly labeled with digoxigenin-11-dUTP (Roche Diagnostics), while the 5S rDNA probes were indirectly labeled with biotin-11-dUTP (Roche Diagnostics, Basel, Switzerland), all by nick translation (ThermoFisher Scientific, Waltham, MA, USA). We followed the manufacturer’s instructions for extraction and labeling procedures.

### 4.3. FISH and Image Processing

For the FISH procedure, we followed Braz et al. [56] with minor modifications. The hybridization mix (10 µL/slide) contained 50% formamide, 10% dextran sulfate, and 2× saline sodium citrate (SSC). Depending on the type of probe, we used different concentrations. For chromosome painting, we added 400 ng of *Pv*2 and 350 ng of *Pv*3 or 300 ng of *Pv*1 and 350 ng of *Pv*5 to the hybridization mix. For the oligo-FISH barcode, we added 200 ng of library 1 and 250 ng of library 2 to the hybridization mix, while for BAC clones, 5S and 35S rDNA, and telomeric probes, we added 10 ng/µL. The final hybridization mix was directly applied to the slides for 7 min at 75 °C and hybridized for 18–36 h at 37 °C. After incubation, the slides were washed with 2× SCC and subsequently with 0.1× SSC at 42 °C, in the absence of formamide (76% stringency). The slides were then incubated for 30 min at 37 °C in 50 µL of 1× TNB (1 M Tris HCl pH 7.5, 3 M NaCl and blocking reagent, Sigma-Aldrich, St. Louis, MO, USA) or 5% BSA. For probe detection, the antibody mix consisted of 0.1–0.2 µL of rhodamine sheep anti-DIG (Roche) and 0.2 µL of Alexa Fluor 488 Streptavidin (Invitrogen, Waltham, MA, USA) diluted up to 20 µL with 1× TNB or 1% BSA and incubated for 1 h at 37 °C. Subsequently, the slides were washed three times with 4× SCC/1% Tween-20, 5 min each at 42 °C, and counterstained with 2 µg/mL DAPI (4,6-diamidino-2-phenylindole) in Vectashield (Vector Laboratories, Newark, CA, USA) antifade solution. To detect different DNA sequences using the same slide, we performed the rehybridization procedure according to Heslop-Harrison et al. [61].

Metaphase images were captured using a CytoVision Station (Leica, Wetzlar, German) with a MV4 camera (Jai, Valby, Denmark) with CytoVision Version 7.7/FISH software upgrade, connected to a Leica DM2500 microscope. The images were adjusted for brightness and contrast using Adobe Photoshop CS6 software. The chromosomes were identified based on their orthology with *V. unguiculata* [25,55]. The measurements were performed for both chromatids of five chromosomes from metaphase plates. The positions of each barcode, rDNA, telomeric, and BAC signal were determined using DRAWID 0.26 software [62]. The idiograms of *V. lasiocarpa* and *V. unguiculata* were performed using Adobe Photoshop CS6 software. *Vigna longifolia* idiogram was adapted from Dias et al. [27].

## 5. Conclusions

This study presents a comprehensive cytogenetic analysis of a dysploid *Vigna* karyotype, offering new insights into the chromosomal structure and evolutionary dynamics of *V. lasiocarpa*. By combining oligo- and BAC-FISH with telomeric DNA probes, we identified key chromosomal rearrangements, including inversions and an “end-to-end chromosome fusion”, supporting the descending dysploidy in this species. Specifically, we propose that a fusion between chromosomes 5 and 7 gave rise to the reduced chromosome number observed in *V. lasiocarpa* (2*n* = 20). This study not only enhances our understanding of the chromosomal structure of *V. lasiocarpa* but also provides insights into the evolutionary mechanisms that have shaped genomic diversity within the *Vigna* genus.

## Data Availability

The original contributions presented in the study are included in the article/Supplementary Material, further inquiries can be directed to the corresponding author.

## Supplementary Materials

The following supporting information can be downloaded at https://www.mdpi.com/article/10.3390/plants14121872/s1: Table S1: List of probes and their chromosomal positions, including barcode^1^, BAC clones^1^, and 5S and 35S rDNA^1,2^, tested and used for comparative FISH mapping among *V. unguiculata*, *V. lasiocarpa* and *V. longifolia*, organized from the terminal region of the short arm to the terminal region of the long arm. These probe sets were used to characterize the *V. lasiocarpa* karyotype and to identify chromosomal rearrangements and homoeology with *V. unguiculata* and *V. longifolia*. The probes’ positions on *V. longifolia* chromosomes are based on Dias et al. [27]. No BACs probes were hybridized to the chromosomes of this species. Hashtags (#) on *Vl*2, *Vla*3, *Vla*4, *Vla*6, *Vl*6, and *Vla*9 indicate changes in chromosome arm orientation compared to *V. unguiculata*. Figure S1: Oligobarcode probes (red and green) hybridized to metaphase chromosomes of *Vigna unguiculata* (a, *Vu*, 2*n* = 22), *V. lasiocarpa* (b, *Vla*, 2*n* = 20), and *V. longifolia* (c, *Vl*, 2*n* = 22). (d) Karyogram showing the barcode pattern on chromosome 3 of the three species, with the centromeres aligned along a thin white line. All chromosomes were counter-stained with DAPI (pseudocolored in gray). Bars in (c,d) = 5 µm.

## Abbreviations

The following abbreviations are used in this manuscript:

FISH	Fluorescence in situ hybridization
BAC	Bacterial artificial chromosome
EMBRAPA	Empresa Brasileira de Pesquisa Agropecuária
NGPS	National Plant Germplasm System
RT	Room temperature
EEF	End-to-end fusion
rDNA	Ribosomal DNA
IPA	Instituto Agronômico de Pernambuco
APK	Ancestral Phaseoleae karyotype
GB	Genomic blocks
EC	Evolutionary new centromeres
DSB	Double-strand breaks
ITR	Interstitial telomeric repeats
SSC	Saline sodium citrate
ChIP-seq	Chromatin immunoprecipitation sequencing
*Pv*	*Phaseolus vulgaris*
*Vu*	*Vigna unguiculata*
*Vl*	*Vigna longifolia*
*Vla*	*Vigna lasiocarpa*
