# Supplementary material for: “End-to-End Chromosome Fusion” as the Main Driver of Descending Dysploidy in Vigna lasiocarpa (Mart. ex Benth.) Verdc. (Leguminosae Juss.)
Source: Plants (Basel). 2025 Jun 18;14(12):1872. doi: 10.3390/plants14121872 (PMC12197010; doi:10.3390/plants14121872)
Supplement: Supplementary file 1 [file plants-14-01872-s001.zip › plants-3698624-supplementary.pdf]

**Table S1.** List of probes and their chromosomal positions, including barcode[1], BAC clones[1], and 5S and 35S rDNA[1,2], tested and used for comparative FISH mapping among *V. unguiculata, V. lasiocarpa* and *V. longifolia*, organized from the terminal region of the short arm to the terminal region of the long arm. These probe sets were used to characterize the *V. lasiocarpa* karyotype and to identify chromosomal rearrangements and homoeology with *V. unguiculata* and *V. longifolia*. Probes positions on *V. longifolia* chromosomes are based on Dias et al. [27]. No BACs probes were hybridized to the chromosomes of this species. Hashtags (#) on *Vl*2, *Vla*3, *Vla*4, *Vla*6, *Vl*6 and *Vla*9 indicate changes in chromosome arm orientation compared to *V. unguiculata*.

| Chr | **Probe/chromosomal position (arm)** | | |
|---|---|---|---|
| | ***V. unguiculata*** | ***V. lasiocarpa*[a]** | ***V. longifolia*[a]** |
| 1 | 35S rDNA/subterminal (S) | Green signal/subterminal (S) | Green signal/subterminal (S) |
| | Red signal/interstitial (S) | H004H23*/subterminal (S) | Green signal/interstitial (S) |
| | Green signal/interstitial (L) | Green signal/interstitial (S) | Green signal/subterminal (L) |
| | H004H23*/subterminal (L) | M50F11*/ interstitial (L) | |
| | Green signal/subterminal (L) | Green signal/subterminal (L) | |
| 2 | 35S rDNA/subterminal (S) | Red signal/interstitial (S) | Red signal/subterminal (L)# |
| | Red signal/interstitial (S) | Green signal/interstitial (L) | 35S rDNA/proximal (L) |
| | Green signal/interstitial (L) | Red signal/subterminal (L) | Green signal/interstitial (S) |
| | Red signal/subterminal (L) | | Red signal/subterminal (S) |
| 3 | Green signal/subterminal (S) | Green signal/subterminal (L)# | Green signal/subterminal (S) |
| | Green signal/interstitial (S) | Green signal/interstitial (L) | Green signal/interstitial (S) |
| | 142D9**/ interstitial (L) | 142D9**/interstitial (L) | Red signal/interstitial (L) |
| | Red signal/interstitial (L) | Red signal/interstitial (L) | Red signal/interstitial (L) |
| | H050P11*/ interstitial (L) | Red signal/interstitial (S) | Red signal/subterminal (L) |
| | Red signal/interstitial (L) | 199D13**/interstitial (S) | |
| | 199D13**/interstitial (L) | Red signal/interstitial (S) | |
| | Red signal/subterminal (L) | H050P11*/ subterminal (S) | |
| 4 | Green signal/subterminal (S) | Green signal/interstitial (L)# | Green signal/subterminal (S) |
| | Green signal/interstitial (S) | Red signal/interstitial (L) | Red signal/interstitial (S) |
| | Red signal/interstitial (L) | Green signal/interstitial (S) | Green signal/interstitial (L) |
| | Red signal/interstitial (L) | Red signal/interstitial (S) | Red signal/interstitial (L) |
| 5 | Green signal/subterminal (S) | | Red signal/interstitial (S) |
| | Green signal/interstitial (S) | | Green signal/interstitial (L) |
| | M50F11*/ interstitial (L) | | Green signal/ interstitial (L) |
| | Green signal/subterminal (L) | | |
| 6 | Red signal/interstitial (S) | Red signal/subterminal (L)# | 35S rDNA/subterminal (S)# |
| | 35S rDNA/subterminal (L) | | Red signal/subterminal (L) |
| 7 | H088A15*/subterminal (S) | | Red signal/proximal (L) |
| | Red signal/interstitial (L) | | Red signal/subterminal (L) |
| | Red signal/interstitial (L) | | |
| 8 | Green signal/ interstitial (S) | Green signal/ interstitial (S) | Green signal/ interstitial (S) |
| | Red signal/interstitial (L) | Red signal/interstitial (L) | Red signal/interstitial (L) |

| | | | |
|---|---|---|---|
| 9 | Red signal/interstitial (S)<br>35S rDNA /proximal (L)<br>Red signal/interstitial (L)<br>H10M18*/subterminal (L) | Red signal/interstitial (L)#<br>Red signal/interstitial (L)<br>H10M18*/subterminal (S) | Red signal/interstitial (S)<br>Red signal/proximal (L) |
| 10 | 5S rDNA/ interstitial (S)<br>Green signal/ interstitial (L)<br>Green signal/ interstitial (L)<br>35S rDNA/subterminal (L) | 35S rDNA/proximal (L)<br>5S rDNA/subterminal (L)<br>Green signal/ interstitial (L)<br>Green signal/ interstitial (L) | 5S rDNA/proximal (L)<br>Green signal/ interstitial (L)<br>Green signal/ interstitial (L) |
| 11 | Green signal/subterminal (S)<br>35S rDNA/interstitial (S)<br>5S rDNA/interstitial (S)<br>Red signal/ interstitial (L)<br>Red signal/subterminal (L) | Green signal/subterminal (S)<br>Red signal/ interstitial (L)<br>Red signal/ interstitial (L) | Green signal/subterminal (S)<br>Red signal/ interstitial (L)<br>Red signal/subterminal (L) |
| *Vla*7/5 | | Red signal/ interstitial (S)<br>Red signal/ interstitial (L)<br>H088A15*/interstitial (L)<br>Red signal/ interstitial (L)<br>Green signal/ interstitial (L)<br>Green signal/subterminal (L) | |

[1] Barcode, BACs and rDNA marks of *V. unguiculata* followed de Oliveira Bustamante et al. [26].
[2] rDNA marks of *V. longifolia* followed Dias et al. [27]
[a] Chromosomes of *V. lasiocarpa* and *V. longifolia* are numbered according to their homoeology with *V. unguiculata*.
* BACs from *V. unguiculata* chromosomes.
** BACs from *Phaseolus vulgaris* chromosomes.
L = long arm; S = short arm.

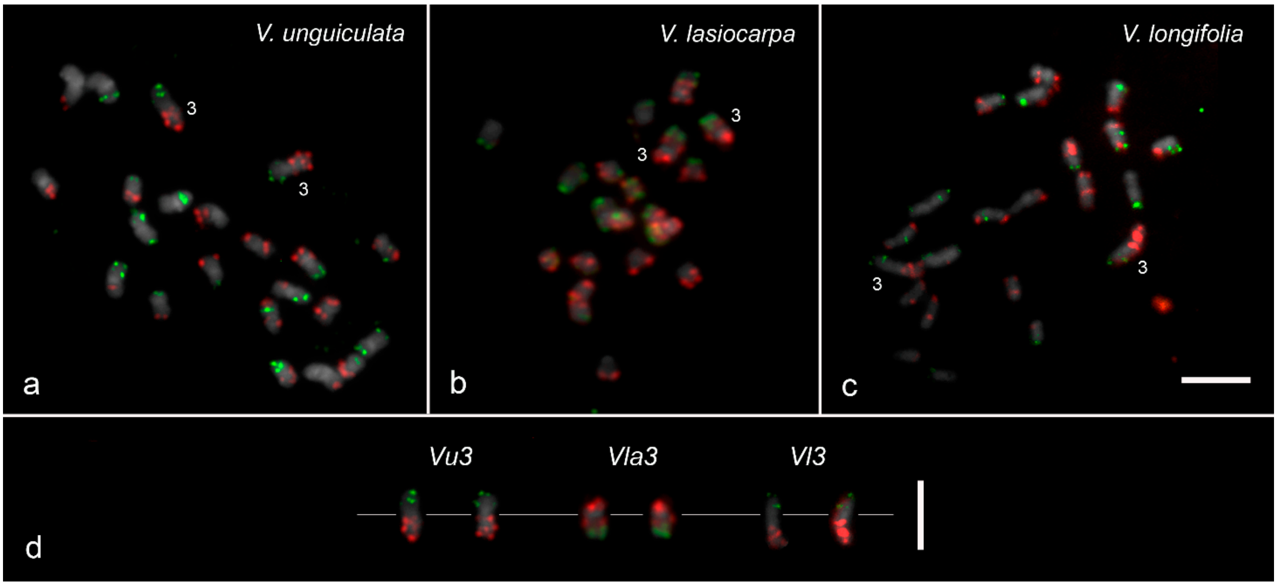


**Figure S1.** Oligobarcode probes (red and green) hybridized to metaphase chromosomes of *Vigna unguiculata* (**a**, *Vu*, *2n* = 22), *V. lasiocarpa* (**b**, *Vla*, *2n* = 20), and *V. longifolia* (**c**, *Vl*, *2n* = 22). (**d**) Karyogram showing the barcode pattern on chromosome 3 of the three species, with the centromeres aligned along a thin white line. All chromosomes were counter-stained with DAPI (pseudocolored in gray). Bars in (**c**) and (**d**) = 5 μm.
